# 17230 Agreement between point-of-care intestinal ultrasound (POCUS) and magnetic resonance enterography for assessment of the terminal ileum through sigmoid colon in pediatric patients with inflammatory bowel diseases: A diagnostic cross-sectional study

**DOI:** 10.1017/cts.2021.660

**Published:** 2021-03-30

**Authors:** Mallory Chavannes, Jonathan R. Dillman, Araz Marachelian, D Brent Polk

**Affiliations:** 1 Children’s Hospital Los Angeles; 2 Cincinnati Children’s Hospital Medical Center; 3 Rady Children’s Hospital San Diego

## Abstract

ABSTRACT IMPACT: Preliminary results will inform the formal evaluation of the reliability of point-of-care ultrasound (POCUS) done by the gastroenterologist compared to standard of care methods such as MR-Enterography. OBJECTIVES/GOALS: Evaluation of mucosal healing is standard for pediatric patients with inflammatory bowel disease (IBD). Point-of-care ultrasound is a non-invasive, cost-efficient tool for assessing intestinal inflammation. We aim to evaluate the agreement between POCUS and typical cross-sectional imaging, such as MR-Enterography (MRE). METHODS/STUDY POPULATION: In this cross-sectional study, we recruited consecutive patients newly diagnosed with IBD, presenting to the specialty outpatient clinic or hospitalized in a pediatric tertiary care center between August to November 2020. They underwent POCUS performed by a single gastroenterologist, in addition to MRE. The sonographer was blinded to MRE results. Bowel wall thickness (BWT) was measured across different bowel segments and recorded twice in longitudinal view and twice in axial view. An average segmental BWT of the four measurements of more than 3 mm was considered inflamed. Agreement between sections of the bowel measured as inflamed were compared to inflamed bowel segments seen by MRE, using Cohen’s kappa. RESULTS/ANTICIPATED RESULTS: Eight of 12 patients completed both MRE and POCUS.A total of 40 bowel segments were assessed, namely the terminal ileum, ascending, transverse, descending and sigmoid colon. There were 4 girls with a median age of 15 years (IQR 14.25-16 years), and 6 patients were diagnosed with Crohn’s disease. Median PCDAI was 32.5 (IQR 30.6-40), and median PUCAI was 75 (72.5-77.5). Agreement between MRE and point-of-care ultrasound was substantial to perfect for the terminal ileum *(Îº= 0.75, 95%CI 0.31-1), transverse colon (Îº= 1, 95%CI 1-1) and sigmoid colon (Îº= 1, 95%CI 1-1). The agreement was poor for the ascending (Îº= 0, 95%CI 0-0) and moderate for the descending colon. (Îº= 0.6, 95%CI -0.07-1) DISCUSSION/SIGNIFICANCE OF FINDINGS: In pediatric patients with IBD, we found a high agreement between POCUS and MRE for imaging of the terminal ileum, transverse and sigmoid colon, areas commonly involved in IBD. This reinforces adult data, outlining the potential of POCUS as an evaluation tool of disease activity in clinical practice.

